# A Novel Strategy of In Situ Trimerization of Cyano Groups Between the Ti_3_C_2_T_*x*_ (MXene) Interlayers for High-Energy and High-Power Sodium-Ion Capacitors

**DOI:** 10.1007/s40820-020-00473-7

**Published:** 2020-06-25

**Authors:** Siyang Liu, Fangyuan Hu, Wenlong Shao, Wenshu Zhang, Tianpeng Zhang, Ce Song, Man Yao, Hao Huang, Xigao Jian

**Affiliations:** 1 School of Materials Science and Engineering, State Key Laboratory of Fine Chemicals, Key Laboratory of Energy Materials and Devices (Liaoning Province), Dalian University of Technology, Dalian, 116024 People’s Republic of China; 2 State Key Laboratory of Fine Chemicals, Department of Polymer Science and Engineering, Dalian University of Technology, Dalian, 116024 People’s Republic of China; 3 School of Mathematical Sciences, Dalian University of Technology, Dalian, 116024 People’s Republic of China

**Keywords:** Sodium-ion capacitors, MXene, Fast kinetics, Triazine polymerization, Nitrogen doping

## Abstract

2D MXenes are attractive for energy storage applications because of their high electronic conductivity. However, it is still highly challenging for improving the sluggish sodium (Na)-ion transport kinetics within the MXenes interlayers. Herein, a novel nitrogen-doped Ti_3_C_2_T_*x*_ MXene was synthesized by introducing the in situ polymeric sodium dicyanamide (Na-dca) to tune the complex terminations and then utilized as intercalation-type pseudocapacitive anode of Na-ion capacitors (NICs). The Na-dca can intercalate into the interlayers of Ti_3_C_2_T_*x*_ nanosheets and simultaneously form sodium tricyanomelaminate (Na_3_TCM) by the catalyst-free trimerization. The as-prepared Ti_3_C_2_T_*x*_/Na_3_TCM exhibits a high N-doping of 5.6 at.% in the form of strong Ti–N bonding and stabilized triazine ring structure. Consequently, coupling Ti_3_C_2_T_*x*_/Na_3_TCM anode with different mass of activated carbon cathodes, the asymmetric MXene//carbon NICs are assembled. It is able to deliver high energy density (97.6 Wh kg^−1^), high power output (16.5 kW kg^−1^), and excellent cycling stability (≈ 82.6% capacitance retention after 8000 cycles).

**Electronic supplementary material:**

The online version of this article (10.1007/s40820-020-00473-7) contains supplementary material, which is available to authorized users.

## Highlights

- A novel N-doped strategy of C_2_N_3_^−^ in situ trimerization between the 2D MXene interlayers was first proposed.
- The ultra-fast pseudocapacitive behavior of Ti_3_C_2_T_*x*_/Na_3_TCM anode was managed and verified.
- The as-fabricated sodium-ion capacitor delivers excellent electrochemical performance by anode/cathode mass matching.

**Electronic supplementary material:**

The online version of this article (10.1007/s40820-020-00473-7) contains supplementary material, which is available to authorized users.

## Graphical abstract

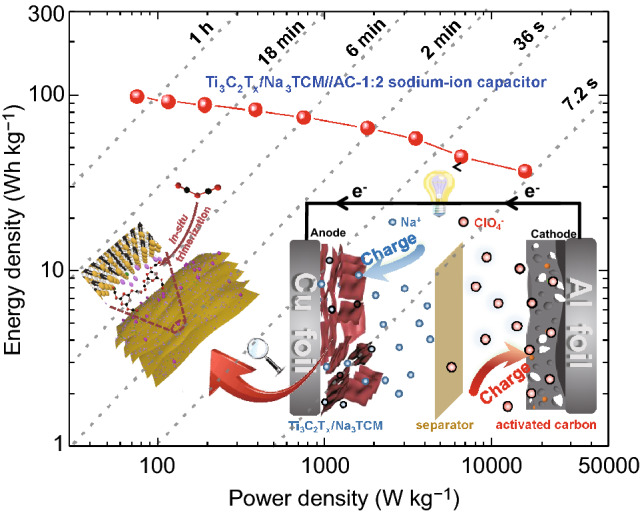

## Introduction

The efficient and price-advantaged energy storage technologies are all important for high-energy and power storage fields, such as communications, smart grids, and electric vehicles [1, 2]. Sodium (Na)-ion energy storage technologies are the most promising alternatives to lithium (Li)-ion energy storage devices due to the similar physical and chemical properties of Na and Li [3]. Additionally, Na has the advantages of being naturally abundant and widely distributed [4]. In particular, Na-ion capacitors (NICs) are emerging rapidly, which can bring the energy density one-step closer towards bridging the battery–supercapacitor divide [5]. However, Na ions have more sluggish transfer kinetics due to their larger radius compared to Li ions (1.02 vs. 0.76 Å) [6]. This disadvantage poses a significant challenge for researchers to develop the NICs that could achieve high energy–power densities and outstanding cycle stability simultaneously [7, 8]. For example, the conventional graphite used for Li-ion battery exhibits a low Na storage capacity of ≈ 35 mAh g^–1^, which is far from enough for the practical applications. Hence, it is urgent to controllable synthesis of novel electrode materials, ensuring rapid diffusion and reversible adsorption/desorption of Na ions during the charging/discharging process.

Two-dimensional (2D) MXenes are a series of carbides and carbonitrides with excellent conductivity of up to 10,000 S cm^–1^, thereby receiving an increasing attention in the fields of energy storage and conversion [9, 10]. Typically, they are synthesized through etching the element “A” (mostly Al or Si element) from the MAX precursor and represented using the general formula M_*n*+1_X_*n*_T_*x*_, where M is transition metals (such as Ti, V, or Nb), X represents C or N, n = 1, 2, or 3, and T_*x*_ stands for –F, –O, and –OH surface functional groups [11–13]. Of more than 30 MXenes, Ti_3_C_2_T_*x*_ was the first one to be discovered [14], which has an obvious layered structure with an initial interlayer spacing of ≈ 9.8 Å. In general, Ti_3_C_2_T_*x*_ MXene is an ideal intercalation-type pseudocapacitive material owing to the abundant as well as tunable terminations T_*x*_ [15], which facilitate faster capacitive response and higher energy storage capacity compared to other carbon-based materials. Theoretical calculations have already proven that the Ti_3_C_2_T_*x*_ with low –F, but high –O surface groups showed higher pseudocapacitive characteristics. For example, using density functional theory (DFT) computations, Zhou et al. demonstrated that the –F terminations induce high ionic diffusion and electronic transfer barriers, thereby decreasing the Li or Na storage capacity of Ti_3_C_2_ immensely [16]. Therefore, engineering high pseudocapacitive heteroatoms (such as N, P, and S) on the surfaces and between the interlayers of Ti_3_C_2_T_*x*_ seems to be a good strategy [17, 18]. In particular, N has the excellent characteristics of electronegativity and pseudocapacitance [3, 19], which could significantly improve the surface electronic and chemical properties of Ti_3_C_2_T_*x*_ electrodes. However, the synthetic strategy based on chemically doped MXenes has not been clearly established. In addition, the N-sources with high toxicity and low boiling point, such as urea and ammonia, may cause inconvenience to production and living [20]. Accordingly, the novel and efficient methods are urgently desired to promote the development and application of MXene materials in energy storage and conversion fields.

Achieving efficient bonding of high electrochemically active polymers with 2D Ti_3_C_2_T_*x*_ MXenes is considered as a promising technology [10, 12]. In particular, the trimerization of small molecules to compounds contained triazine ring structure is commonly known in the chemical process of carbonitrides [21], including the famous trimerization reaction of cyanamide (CH_2_N_2_) to melamine (C_3_H_6_N_6_). As early as 1922, the trimerization of dicyanamide anions (C_2_N_3_^−^ or N(CN)_2_^−^) to tricyanomelaminate ions (C_6_N_9_^3−^) through the heating method in solid phase was reported [22]. To the best of our knowledge, no follow-up studies of the C_2_N_3_^−^ anionic trimerization were reported in the energy storage field. Sodium dicyanamide (Na-dca) is the commercially available product, which can be applied to fabricate organic superconductors or synthesize heterocyclic compounds [23]. Na-dca could intercalate, covalently engineer, and in situ polymerize to sodium tricyanomelaminate (Na_3_TCM) between the Ti_3_C_2_T_*x*_ interlayers, which simultaneously acts as intercalant and N-source. Na-dca as the N-source has low toxicity and light pungent smell compared to urea and melamine [20]. More importantly, the sp^2^-hybridized triazine rings provide high as well as stable N atoms, which are connected by the strong covalent bonds for ensuring the stability during electrochemical cycles [19]. Thus, we proposed a reliable and controllable strategy for achieving highly N-doped Ti_3_C_2_T_*x*_ through a highly novel method of the in situ trimerization of cyano groups (C≡N).

In this work, we utilized a one-step strategy toward endowing a modified Ti_3_C_2_T_*x*_ (Ti_3_C_2_T_*x*_/Na_3_TCM) anode for NICs. The as-obtained Ti_3_C_2_T_*x*_/Na_3_TCM exhibited a large interlayer spacing (12.6 Å) and high N-doping (5.6 at.%). The DFT calculations and kinetic analysis have proved that the doped N atoms reduce the Na adsorption energy and Na-ion diffusion kinetic barriers; and the ultra-fast Na-ion storage kinetics was achieved by the increased pseudocapacitance. As anode for NICs, the Ti_3_C_2_T_*x*_/Na_3_TCM showed a high and reversible capacity of 243.4 mAh g^−1^ at 20 mAg^−1^ and an ultra-cycle stable capacity of 182 mAh g^−1^ after 1000 cycles at 0.1 A g^−1^. Basing on the charge balance principle, the Ti_3_C_2_T_*x*_/Na_3_TCM anode was well matched with activated carbon (AC) cathode in the different mass ratios (1:1, 1:2, 1:3) for achieving better charge-transfer kinetics. The NICs with the anode/cathode mass ratio of 1:2 (Ti_3_C_2_T_*x*_/Na_3_TCM//AC-1:2) delivered high energy and power densities (97.6 Wh kg^−1^ at 76 W kg^−1^ and 36.6 Wh kg^−1^ at 16.5 kW kg^−1^) as well as ultra-stable cycling life within 0–4.0 V (≈ 82.6% capacitance retention after 8000 cycles).

## Experimental

### Synthesis of Ti_3_C_2_T_***x***_ MXene

Ti_3_C_2_T_*x*_ was synthesized by treating 2 g of Ti_3_AlC_2_ (11 Technology Co., Ltd.) in 40 mL of 40% HF aqueous solution for 24 h at 30 °C and 200 rpm. The resulting solution was centrifuged at 4000 rpm to remove the impurities and residual HF until the pH reached 6–7. Finally, the obtained powders were dried in vacuum at 60 °C for 24 h.

### Synthesis of Ti_3_C_2_T_***x***_/Na_3_TCM Composite

Ti_3_C_2_T_*x*_/Na_3_TCM composite was prepared by a simple hydrothermal for the thermal trimerization of sodium dicyanamide (Na-dca) to sodium tricyanomelaminate (Na_3_TCM) between Ti_3_C_2_T_*x*_ interlayers. In a typical synthesis, 200 mg of Na-dca (Aladdin, 98%) was dissolved in 40 mL of deionized water. After that, 200 mg of Ti_3_C_2_T_*x*_ was dispersed in the as-obtained Na-dca solution and stirred at room temperature at 400 rpm for 2 h. Then, the mixture was sealed in the N_2_ atmosphere and sonicated in an ice bath at 250 W for 1 h to produce a homogeneous solution. Subsequently, the mixed solution was transferred to a Teflon-lined autoclave and the temperature was raised to 180 °C at a heating speed of 2 °C and then maintained for 6 h. The resulting powder was washed several times with deionized water during vacuum filtration and then dried at 60 °C in the vacuum oven.

### Materials Characterization

FT-IR spectra were recorded with a Thermo Fisher 6700 spectrometer by using the KBr pellet method. XRD patterns were obtained with a Rigaku D/Max 2400 diffractometer by using Cu Kα radiation operated at 45 kV and 200 mA. Nitrogen sorption/desorption isotherms were characterized at 77 K with a Micromeritics ASAP 2020 analyzer. Prior to the gas adsorption, powders were outgassed at 120 °C for 8 h under a vacuum of 10^−6^ Torr. To further characterize the structure, the as-prepared samples were observed and characterized using the SEM (Hitachi, SU8220) and HR-TEM (FEI, Eindhoven, The Netherlands). In addition, XPS measurements were measured using an ESCALAB 250 analyzer with an Al Kα X-ray source and a base pressure of 1 × 10^−9^ mbar.

### Electrochemical Measurement

The working electrodes (Ti_3_C_2_T_*x*_ and Ti_3_C_2_T_*x*_/Na_3_TCM anode, AC cathode) were prepared by mixing the active material (80 wt%), polyvinylidene fluoride (10 wt%), and acetylene black (10 wt%) in N-methyl-2-pyrrolidone (NMP) solvent. The obtained mixtures were coated on the Cu foil for anode or Al foil for cathode and then dried at 80 °C in vacuum oven for 12 h. The loading and diameter of working electrode were 1.6–1.8 mg cm^–2^ and 11 mm, respectively. The electrolyte was 1 M NaClO_4_ in ethylene carbonate, diethyl carbonate (EC/DEC, v/v = 1:1) with 5 vol% fluoroethylene carbonate (FEC) as the electrolyte additive, and the glass microfiber GF/D was used as separator. For half-cell, the working electrodes were assembled with metallic Na in a CR2032 coin-type cell. For NICs, the Ti_3_C_2_T_*x*_/Na_3_TCM anode was discharged and charged for 10 cycles in a half-cell at 0.1 A g^−1^ and then assembled with the YP80F AC cathode in the mass ratios of 1:1, 1:2, 1:3 in the CR2032 coin-type cell. All cells were assembled and disassembled inside a glove box with sub 0.1 ppm water and oxygen contents. The electrochemical performances were evaluated using the Land CT2001A testers system and Biologic VMP3 electrochemical workstation. The EIS was performed at the frequency range from 100 kHz to 0.01 Hz. The energy density *E* (Wh kg^−1^) and power density *P* (W kg^−1^) were calculated using Eqs. 1 and 2 [24, 25]:

1 $$E={\int }_{t1}^{t2}IV\mathrm{d}t$$

2 $$P=E/t$$

where *I* (A g^−1^) is the specific discharge current based on the total mass of active material on both electrodes, *V* (V) is the discharge voltage, and *t* (s) is the discharge time.

### Calculating Details

The DFT calculations were carried out using the projector-augmented wave pseudopotentials as implemented in the Vienna ab initio simulation package (VASP) [26]. Exchange correlation interactions were described by the Perdew–Burke–Ernzerhof generalized gradient approximation (GGA) [27]. Since the dipole and van der Waals (vdW) effects on total energy were small, no dipole and vdW corrections were considered. The plane wave kinetic energy cutoff was set to be 500 eV. Atomic positions and lattice parameters were fully relaxed at the GGA level until the atomic forces were smaller than 0.01 eV Å^−1^. The convergence criteria for structural optimization was set to be 10^−5^ eV in energy. The Na-ion diffusion barriers on the Ti_3_C_2_T_*x*_ surface were calculated using the climbing image nudged elastic band (CI-NEB) method [28]. A 1 × 1 × 1 unit cell was used to evaluate the adsorption properties of O and F atoms on Ti_3_C_2_ surface. A 3 × 3 × 1 supercell was used to investigate the diffusion barrier of Na ion. The Brillouin zone was represented by Monkhorst–Pack special k-point mesh of 12 × 12 × 1 for the unit cell and 4 × 4 × 1 for supercell calculations, respectively. The adsorption energy (*E*_ads_) of the atoms and adsorbates was calculated according to the following equation: *E*_ads_ = *E*_total_ – *E*_adsorbates_ – *E*_a_, where *E*_total_, *E*_adsorbates_, and *E*_a_ are the total energies of the relaxed adsorption system, the various adsorbates, and the adsorbed atom (O, F, or Na), respectively.

## Results and Discussion

### Structure Characterization

**Fig. 1:**
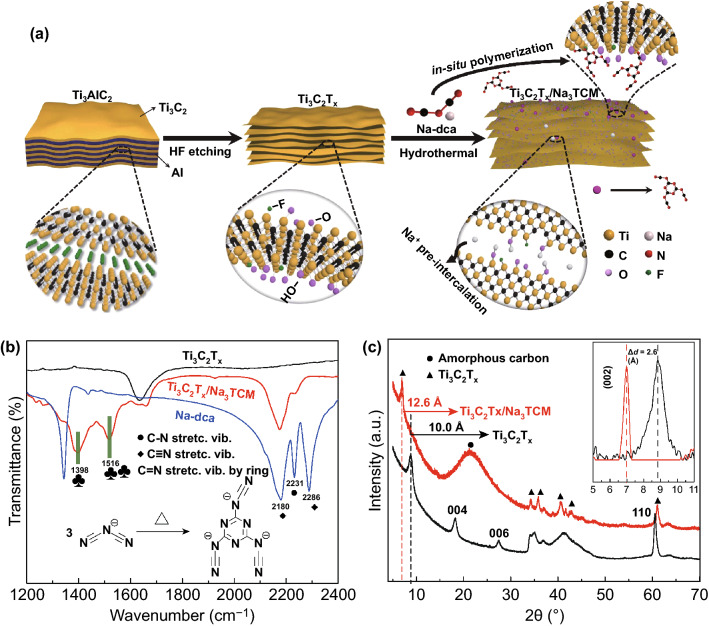
**a** Schematic illustration of the synthesis for the Ti_3_C_2_T_*x*_/Na_3_TCM. **b** FTIR spectra of Na-dca, Ti_3_C_2_T_*x*_, and Ti_3_C_2_T_*x*_/Na_3_TCM; inset shows the thermal trimerization process of C_2_N_3_^−^ to C_6_N_9_^3−^. Peak labels correspond to (black circle) 2231 cm^–1^ *v*_s_C–N + *v*_as_C–N, (black diamond) 2286 cm^–1^ *v*_s_C≡N and 2180 cm^–1^ *v*_as_C≡N, (black club suit) 1398 and 1516 cm^–1^ *v*_as_Ring N + *v*_s_Ring N. **c** XRD patterns of Ti_3_C_2_T_*x*_ and Ti_3_C_2_T_*x*_/Na_3_TCM; inset shows the magnification of XRD patterns. Peak labels correspond to (black circle) amorphous carbon and (black triangle)Ti_3_C_2_T_*x*_

The synthetic scheme of Ti_3_C_2_T_*x*_/Na_3_TCM nanosheets is illustrated in Fig. 1a; the accordion-like Ti_3_C_2_T_*x*_ was produced by selective etching of the Al element from precursor Ti_3_AlC_2_ [29, 30]. The obtained Ti_3_C_2_T_*x*_ was dispersed in the Na-dca solution and followed with stirring and ultrasonic processes under N_2_ atmosphere. Afterward, the C_2_N_3_^−^ ions trimerize to form C_6_N_9_^3−^ ions during the hydrothermal process, resulting in stable and high N-doping effect. Meanwhile, the C_6_N_9_^3−^ ions were covalently engineered between the Ti_3_C_2_T_*x*_ interlayers by breaking sectional Ti–F bonds and the electrostatic interactions during solvent evaporation [28]. The successful trimerization of C_2_N_3_^−^ ions (Fig. 1b, inset) was confirmed based on the Fourier-transform infrared (FT-IR) spectra. The vibration peaks at 2180 and 2286 cm^–1^ decrease sharply due to the formation of C_6_N_9_^3−^ ions as shown in the red spectrum in Fig. 1b. In an opposite manner, two strong peaks at about 1398 and 1516 cm^–1^ appear, indicating the vibration of triazine/benzene structures [31, 32]. The peaks at 1635 and 1670 cm^−1^ could be attributed to the C–O and C=O bonds, respectively, and the small peak at 1320 cm^−1^ corresponds to the stretching vibration of C–N bond [33]. It is noteworthy that the C_6_N_9_^3−^ consists of a triazine ring with three N≡C–N. The ring has no threefold symmetry because of the turning of one of the N≡C–N sidearms, preventing the further long-chain polymerization [21]. Furthermore, interestingly, C_2_N_3_^−^ ions were trimerized without catalysts during the entire work, which may be attributed to the functional groups or the interstratified pressure of Ti_3_C_2_T_*x*_. This advantage contributes to the cost saving and the environmental protection.

To further investigate the interlayer spacing and the crystalline phase of Ti_3_C_2_T_*x*_/Na_3_TCM, the X-ray diffraction (XRD) patterns were carried out (Fig. 1c). A broad peak centered at around 23° can be observed in Ti_3_C_2_T_*x*_/Na_3_TCM, which is assigned to the scattering of amorphous carbon derived from trimerization products [31, 32, 34, 35]. Besides, no supererogatory diffraction peaks are found after introducing Na_3_TCM, confirming the successful synthesis of Ti_3_C_2_T_*x*_/Na_3_TCM composite. The Ti_3_C_2_T_*x*_ could survive throughout the hydrothermal reactions without appearing damage in morphology and texture mainly due to the protection of nanoplating of Na_3_TCM [36]. Interestingly, the (002) peak moves toward a lower diffraction angle from 8.8° in Ti_3_C_2_T_*x*_ to 6.8° in Ti_3_C_2_T_*x*_/Na_3_TCM, and the average interlayer spacing can be calculated by the center position of (002) peaks using the Bragg formula [37]. Ti_3_C_2_T_*x*_/Na_3_TCM has a larger average interlayer spacing of 12.6 Å compared to Ti_3_C_2_T_*x*_ of 10.0 Å, allowing for additional intercalation and adsorption of Na ions. In addition, we used theoretical calculations combined with experimental result to illustrate the existence of dca^−^ and TCM^3−^ ions between the Ti_3_C_2_T_*x*_/Na_3_TCM MXene interlayers. The schematic drawing about the size of dca^−^ and TCM^3−^ ions and the widths of single Ti_3_C_2_ layer are presented in Fig. S1, which are obtained by the DFT calculation. Since the interlayer spacing of Ti_3_C_2_T_*x*_/Na_3_TCM is 1.26 nm and the thickness of single Ti_3_C_2_ layer is 0.47 nm, a slant manner of intercalated dca^−^ and TCM^3−^ ions to the broad plane is considered. Hence, the expansion of interlayer spacing is attributed to the combined effects of polymerized C_6_N_9_^3−^ ions and dissociated Na^+^ ions [38], thereby providing an open spacing and avoiding the stacking and stability issues of 2D nanosheets.

**Fig. 2:**
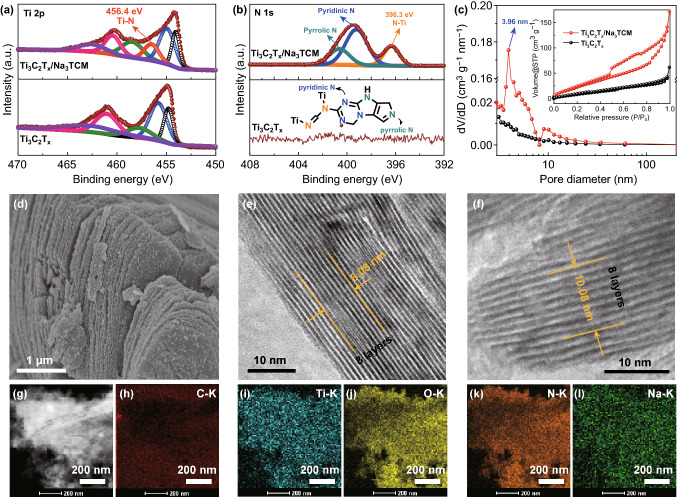
Characterization of Ti_3_C_2_T_*x*_/Na_3_TCM and Ti_3_C_2_T_*x*_. **a** High-resolution Ti 2p XPS spectra of Ti_3_C_2_T_*x*_/Na_3_TCM (top) and Ti_3_C_2_T_*x*_ (bottom). **b** High-resolution N 1s XPS spectra of Ti_3_C_2_T_*x*_/Na_3_TCM (top) and Ti_3_C_2_T_*x*_ (bottom); insets in bottom show the schematic illustration of N functional groups. **c** Pore size distributions of Ti_3_C_2_T_*x*_/Na_3_TCM and Ti_3_C_2_T_*x*_; inset shows the nitrogen adsorption–desorption isotherms. **d** Top-view SEM image of Ti_3_C_2_T_*x*_/Na_3_TCM. HR-TEM images of **e** Ti_3_C_2_T_*x*_ and **f** Ti_3_C_2_T_*x*_/Na_3_TCM. **g** STEM image of Ti_3_C_2_T_*x*_/Na_3_TCM and the corresponding elemental mapping images of **h** C, **i** Ti, **j** O, **k** N and **l** Na

X-ray photoelectron spectroscopy (XPS) analysis was implemented to illustrate the interaction between intercalated atoms and Ti_3_C_2_T_*x*_ matrix (Fig. S2a). The Ti 2p XPS spectrum of Ti_3_C_2_T_*x*_ is complicated to define due to the various Ti oxidation states. Nevertheless, we can easily observe that the Ti 2p deconvolution peak breaks up into two sublevels by spin–orbit coupling, and the peak centered at 456.4 eV is fitted and assigned to Ti–N (Fig. 2a) [39]. Interestingly, the peak areas and positions of various Ti oxidation states are slightly changed due to the effective combination of Ti and C_6_N_9_^3−^ ions. The content of N atom increases from 0.0 to 5.6 at.% due to the trimerization of C_2_N_3_^−^ (Table S1). N 1s XPS spectrum of Ti_3_C_2_T_*x*_/Na_3_TCM can be fitted with three components for pyrrolic N (21.7%), pyridinic N (55.6%), and N–Ti (22.7%) at the binding energies of 400.6, 399.2, and 396.3 eV, respectively (Fig. 2b) [40]. The nearly 80% of pyrrolic N and pyridinic N derived from triazine rings could rapidly store Na ions and provide high pseudocapacitance [3, 32]. This result proves that C_6_N_9_^3−^ is synthesized, meanwhile covalently bonded with the Ti atom. To further confirm the interior covalent interactions of N and Ti atoms, the depth profiling of XPS was displayed by using the Ar-ion sputtering method (Fig. S3) [41]. After 62 and 124 s of sputtering, the position and shape of N 1s and Ti 2p spectra were not changed, further confirming the strong covalent bonds between N and Ti atoms. On the other hand, the –F termination on MXene surface could benefit the formation of stable SEI layer [42]. However, vast –F terminations induce high ionic diffusion barrier, while not participate in any pseudocapacitive energy storage processes [16, 43]. Hence, the MXenes with more –O terminal groups and fewer –F terminal groups can be expected as electrodes. In Ti_3_C_2_T_*x*_/Na_3_TCM, the intensity of the Ti–F peaks decreased quickly and the contents of N increased significantly in XPS analysis (Figs. S2b and 2b), indicating that large amount of –F terminal groups was replaced by C_6_N_9_^3−^ ions. It is because that the Ti–F bond is easily broken during hydrothermal process, and the electronegativity of N and F is similar. In addition, the C 1s XPS spectra were investigated to clarify the structure of the composite (Fig. S2c). Notably, a redshifting of about 0.3 eV could be observed for the C–Ti bonding in Ti_3_C_2_T_*x*_/Na_3_TCM due to the changed Ti atomic environment. The N=C–N in triazine rings in Ti_3_C_2_T_*x*_/Na_3_TCM confirmed the formation of Na_3_TCM, and the C–Ti–N at 287.1 eV further proved the covalent interactions of N and Ti atoms. Importantly, the Na 1s peak centered at ≈ 1071 eV indicates the pre-intercalated Na ions, which is similar to that of alkalized MXenes (Fig. S2d) [8, 44]. These Na ions play the role of pillar effect and make up for the irreversible capture of Na ions during electrochemical cycling, which will be discussed below.

To provide another evidence of pillar effect, the pore structure and specific surface area analysis were carried out. Observed from the nitrogen adsorption–desorption isotherms, Ti_3_C_2_T_*x*_/Na_3_TCM exhibits the type IV isotherm with a hysteresis loop at the relative pressure from 0.5 to 1.0, indicating the coexistence of micropores and mesopores (Fig. 2c) [45]. Ti_3_C_2_T_*x*_/Na_3_TCM has a large average pore size of ≈ 4 nm, which indicates that C_6_N_9_^3−^ ions were obtained between the Ti_3_C_2_T_*x*_ interlayers. Such “generous” pore structure is essential to achieve high-rate pseudocapacitive energy storage in the 2D nanosheets by accelerating rapid Na-ion diffusion kinetics [46]. Also, Ti_3_C_2_T_*x*_/Na_3_TCM possesses a Brunauer–Emmett–Teller specific surface area of 183.4 m^2^ g^−1^, which is nearly 6 times higher than that of the pristine Ti_3_C_2_T_*x*_ (32.5 m^2^ g^−1^). The increased surface area comes from the contribution of highly N-doped trimerization products, which might increase the electrolyte-accessible areas and active sites.

As shown in Figs. 2d and S4, obviously, Ti_3_C_2_T_*x*_/Na_3_TCM exhibits a well-preserved 2D nanosheet structure similar to that of pristine Ti_3_C_2_T_*x*_. Discriminatively, the trimerization products on the Ti_3_C_2_T_*x*_ surfaces provide abundant active surface, which is well matched with the pore structure analysis. Combining the high-resolution TEM (HR-TEM) images with corresponding patterns [47], the average interlayer spacing of the 2D nanosheet increases from 10.1 Å of Ti_3_C_2_T_*x*_ to 12.6 Å of Ti_3_C_2_T_*x*_/Na_3_TCM (Figs. 2e, f and S5), agreeing well with the XRD results. The STEM image and corresponding elemental maps illustrate the spatial distribution of C, Ti, O, N, and Na elements of Ti_3_C_2_T_*x*_/Na_3_TCM at the nanometer scale (Fig. 2g–l). It can be clearly seen that N (orange color) has the same distribution as the even distributed Ti and O elements, demonstrating that C_6_N_9_^3−^ ions are anchored and/or fastened on the Ti_3_C_2_T_*x*_ matrix. However, Na (green color) displays a wide range covering on the Ti_3_C_2_T_x_ matrix like the C element. It can be hypothesized that besides the preformation of –ONa, = NNa on the Ti_3_C_2_T_*x*_ surface, abundant Na ions exist in the secondary piled structure of 2D Ti_3_C_2_T_x_, which could also be considered as the pre-intercalated Na ions. These pre-intercalated Na ions make up for the irreversible and captured Na ions derived from the electrolyte, which might be combined with defects and heteroatoms during the desodiation process, thereby assisting the improvement in Na storage capacity and cycle stability [44, 48]. Furthermore, a series of complicated Moiré patterns are displayed in the HR-TEM image of Ti_3_C_2_T_*x*_/Na_3_TCM (Fig. S6) [49], indicating the multilayered heterostructure of polymerized C_6_N_9_^3−^ stacking on the Ti_3_C_2_T_x_ matrix.

### Electrochemical Performance Tests in Half-Cell

**Fig. 3:**
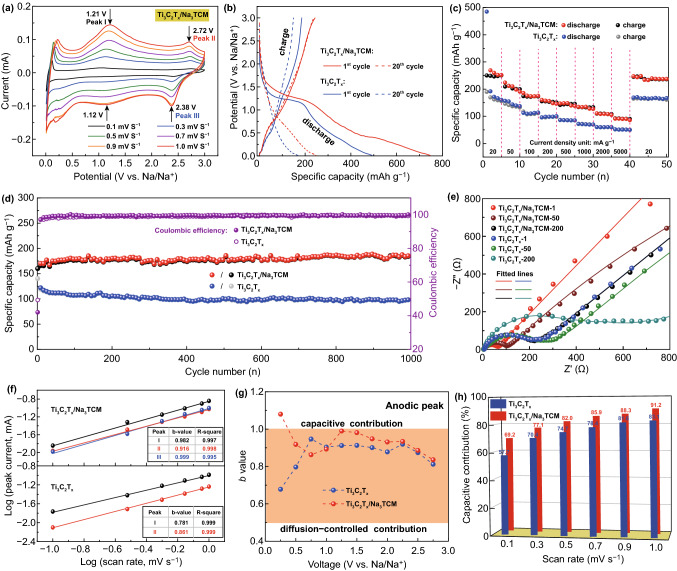
Electrochemical performance and kinetic analysis of Ti_3_C_2_T_*x*_/Na_3_TCM and Ti_3_C_2_T_*x*_ for Na-ion storage. **a** CV curves of Ti_3_C_2_T_*x*_/Na_3_TCM at different scan rates from 0.1 to 1 mV s^−1^. **b** Charge–discharge curves of the 1^st^ cycle and the 20^th^ cycle at the current density of 20 mA g^−1^. **c** Rate performance at various current densities from 20 mA g^−1^ to 5000 mA g^−1^. **d** Long-term cycling performance and the coulombic efficiency at the current density of 100 mA g^−1^. **e** Nyquist plots of Ti_3_C_2_T_*x*_ − 1, Ti_3_C_2_T_*x*_ − 50, Ti_3_C_2_T_*x*_ − 200 and Ti_3_C_2_T_*x*_/Na_3_TCM − 1, Ti_3_C_2_T_*x*_/Na_3_TCM − 50, Ti_3_C_2_T_*x*_/Na_3_TCM − 200. **f** The determination of the *b* values at cathodic and anodic peaks regimes of the Ti_3_C_2_T_*x*_/Na_3_TCM (top) and the Ti_3_C_2_T_*x*_ (bottom). **g** The *b* values of anodic peaks at the different potentials. **h** Capacitive contribution ratios at various scan rates from 0.1 to 1 mV s^−1^

The Na-ion storage performance was investigated by using the coin-type half-cells in the potential window of 0.01–3.0 V (vs. Na/Na^+^). According to the first three cyclic voltammetry (CV) curves of Ti_3_C_2_T_x_ and Ti_3_C_2_T_*x*_/Na_3_TCM, the cathodic peaks at the range of 0.2–1.75 V of the first CV curve show the formation of SEI film or the decomposition of electrolyte (Fig. S7a, b) [50]. And the nearly overlapped second and third CV curves prove the good cycle stability. Interestingly, a symmetric anodic/cathodic peak shown at ≈0.09 V of Ti_3_C_2_T_*x*_/Na_3_TCM indicates the reversible desodiation and sodiation process, respectively. The cyclic stability is therefore improved, because the pre-intercalated Na ions supplement the Na ions derived from the electrolyte, which are irreversible combined with the defects and heteroatoms during the desodiation process. However, for the Ti_3_C_2_T_*x*_, no anodic peaks are exhibited to correspond to the sharp cathodic peak. The current responses in CV tests were recorded at scan rates from 0.1 to 1.0 mV s^−1^ (Figs. 3a and S7c). The shape of the CV curves for the two electrodes is well preserved as the increasing scan rates, indicating good rate capability and small electrode polarization. The Ti_3_C_2_T_*x*_/Na_3_TCM shows two redox couples approximately at 1.21/1.12 and 2.72/2.38 V, which are assigned to the following additional reactions [49, 51]: (1) pseudocapacitive (de)adsorption of Na ions on the N-rich surface; (2) (de)intercalation of Na ions into the nanovoids.

Observed from the galvanostatic charge/discharge (GC/D) curves at the current density of 20 mA g^−1^ (Fig. 3b), Ti_3_C_2_T_*x*_/Na_3_TCM and Ti_3_C_2_T_*x*_ exhibit initial discharge/charge capacities of 746.8/247.7 and 493.1/185.6 mAh g^−1^, resulting in the low coulombic efficiency of ≈ 35% for the first cycle. Such low coulombic efficiency at initial charge/discharge cycle could be also obtained in the previously reported 2D MXene materials, such as Ti_2_CT_*x*_ [52] and V_2_CT_*x*_ [53]. The irreversible capacity loss is most likely caused by the formation of SEI film or the decomposition of electrolyte, which is consistent with the results obtained by CV tests. However, after a few initial cycles, the reversible discharge capacities (the 20th cycle) of 243.4 and 148.2 mAh g^−1^ were exhibited in Ti_3_C_2_T_*x*_/Na_3_TCM and Ti_3_C_2_T_*x*_, respectively. In addition, Ti_3_C_2_T_*x*_/Na_3_TCM has superior high-rate capacities of 210, 174, 157, 147, 135, 109, and 95 at 50, 100, 200, 500, 1000, 2000, and 5000 mA g^−1^, respectively (Fig. 3c). The excellent electrochemical performance is attributed to the enlarged interlayer spacing, open porosity, and N-doping. To further investigate the cycling stability, the two electrodes were evaluated at 100 mA g^−1^ for long cycling (Fig. 3d). It is impressive that the Ti_3_C_2_T_*x*_/Na_3_TCM retains a reversible capacity of 182.2 mAh g^−1^ (≈ 112% of the initial value) after 1000 cycles, which is superior to the Ti_3_C_2_T_*x*_ of only 97.6 mAh g^−1^ after 1000 cycles. The specific capacity of Ti_3_C_2_T_*x*_ decreases sharply during the first 40 cycles, which is caused by the irreversible capture of Na ions by the defects. In comparison, Ti_3_C_2_T_*x*_/Na_3_TCM exhibits a sustaining and growing capacity, which entirely comes from the contribution of pre-intercalated Na ions. This result further demonstrates that the symmetric anodic/cathodic peaks appear in the CV curve of Ti_3_C_2_T_x_/Na_3_TCM rather than Ti_3_C_2_T_*x*_, and the mapping relation of C and Na atoms is also confirmed. Further, the coulombic efficiency of the two electrodes gradually stabilizes at ≈ 99% after the first few cycles. Undeniably, the difference in electrochemical performances is directly related to the microstructure and composition, which become the conclusive factors for charge storage. We have proved that the trimers coated and Na-ion pre-intercalated Ti_3_C_2_T_x_ could markedly facilitate the Na-ion storage.

Electrochemical impedance spectroscopy (EIS) was utilized to reveal the charge transfer characteristics. Nyquist plots consist of a semicircle at high to middle frequencies region and an inclined line at low frequency, which is associated with the ionic diffusion (Fig. 3e) [54]. Basing on the equivalent circuit model (Fig. S8), these points were fitted using the solid lines in the same color (Table S2). For the Ti_3_C_2_T_*x*_/Na_3_TCM, the value of *R*_ct_ corresponds to the semicircle and increases from 6.25 (after 1 cycle) to 21.81 (after 50 cycles) and then to 172.1 Ω (after 200 cycles), which may be caused by the deterioration on the surface of materials during cycling [55]. However, the value of *R*_ct_ of Ti_3_C_2_T_*x*_ increases from 170.4 (after 1 cycle) to 223.5 (after 50 cycles) and then to 365.9 Ω (after 200 cycles), indicating that the Ti_3_C_2_T_*x*_/Na_3_TCM has superior structure for ions transport and electrons transfer. The reasons come from the contribution of the increased N-rich sites and the enlarged interlayer spacing.

To qualitatively evaluate the capacitive contribution, we analyzed the kinetics behaviors of Na-ion storage according to the mathematical equation of $i=a{v}^{b}$. Here, *v* is the scan rate, *a* is a constant, and *b* is an adjustable parameter that ranges from 0.5 (diffusion-controlled process) to 1 (surface-controlled or capacitive process) [56]. The *b* value is much closer to 1, suggesting the larger capacitive contribution to total capacity storage. When *b* is closer to 0.5, diffusion control dominates. High proportion of capacitive contribution facilitates the fast Na-ion storage and promote ultra-high-rate performance [3, 8]. The *b* values of the oxidation and reduction peaks in the Ti_3_C_2_T_*x*_/Na_3_TCM are closer to 1 than that in the Ti_3_C_2_T_x_ (Fig. 3f), suggesting the ultra-fast Na-ion storage kinetics of Ti_3_C_2_T_*x*_/Na_3_TCM governed by the capacitive process. Even the potentials are far from the anodic peak potentials; the *b* values of Ti_3_C_2_T_*x*_/Na_3_TCM and Ti_3_C_2_T_x_ both fluctuate within the range of 0.5–1 (Fig. 3g). This finding suggests that the total capacity storage is the combination of capacitive and diffusion-controlled processes. According to the power-law relationship, the specific capacity can be quantificationally divided into the capacitive (*k*_1_*v*) effects and diffusion-controlled behavior (*k*_2_*v*^1/2^) as follows [57]: $i\left(V\right)={k}_{1}v+{k}_{2}{v}^{1/2}$ (Figs. S9 and S10). The capacitive contribution gradually raises with the increasing scan rates (Fig. 3h), and the maximal capacitive contribution ratio of 91.2% vs. 83.1% (Ti_3_C_2_T_*x*_/Na_3_TCM vs. Ti_3_C_2_T_*x*_) could be obtained at the scan rate of 1.0 mV s^−1^. In general, the capacitive capacity is provided by the electrical double-layer capacitive (EDLC) one and the pseudocapacitive one. The EDLC materials generally have a high specific surface area up to 2000 m^2^ g^−1^ due to the proportional relationship of specific capacitance and specific surface area [56]. As is well known, multilayer Ti_3_C_2_T_*x*_ MXene is the representative pseudocapacitive electrode with a low specific surface area of only ≈50 m^2^ g^−1^. Thus, the capacitance-controlled capacities of Ti_3_C_2_T_*x*_/Na_3_TCM and Ti_3_C_2_T_*x*_ electrodes are basically from the pseudocapacitive contribution. This finding strongly reveals that the electrolyte could be easily accessible to the enlarged interlayer spacing and modified surface of Ti_3_C_2_T_*x*_/Na_3_TCM, promoting the ultra-fast Na-ion storage kinetics.

### First-Principles Calculations

**Fig. 4:**
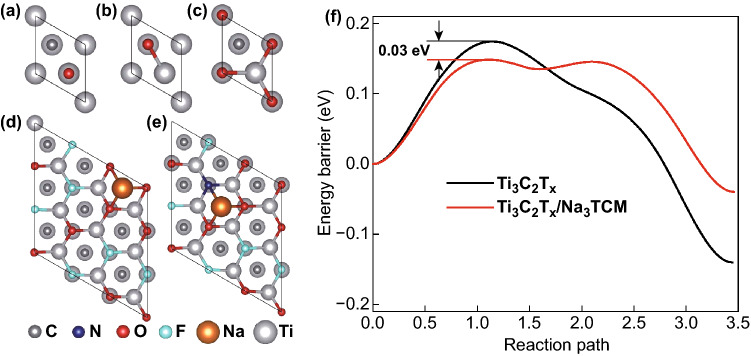
DFT calculations for the Na-ion storage on the Ti_3_C_2_T_*x*_/Na_3_TCM and Ti_3_C_2_T_*x*_ surface. Top view of an O atom adsorbed on 1 × 1 Ti_3_C_2_ surface at **a** top site, **b** bcc site and **c** fcc site. Top view of a Na adsorbs on the most stable site on 3 × 3 surface of **d** Ti_3_C_2_T_*x*_ and **e** Ti_3_C_2_T_*x*_/Na_3_TCM. **f** Diffusion profiles of the Na on Ti_3_C_2_T_*x*_ and Ti_3_C_2_T_*x*_/Na_3_TCM surface in the NEB calculations

The first-principles calculations were performed to present a clear understanding for the remarkable Na storage capacity of N-rich Ti_3_C_2_T_*x*_ surface. First, it is obvious that the O atom (Fig. 4a–c) and F atom (Fig. S11) both prefer to occupy the face-centered cubic (fcc) site to form the Ti_3_C_2_O and Ti_3_C_2_F structures (Table S3). The O and F atomic adsorption energies on the 1 × 1 Ti_3_C_2_ surface occupied at fcc site are − 9.74 and − 7.20 eV, respectively. Then, a 3 × 3 Ti_3_C_2_OF structure was constructed to focus on the adsorption of Na as shown in Fig. S12. After the calculations of all possible Na-adsorbed sites on the Ti_3_C_2_OF surface, the most stable Na-adsorbed fcc site was obtained (Fig. 4d). According to the results obtained from XPS, the increased N ratio is approximate to the decreased F ratio on the Ti_3_C_2_T_*x*_ surface. Therefore, a Ti_3_C_2_T_x_/Na_3_TCM model in the form of the substitute of a N atom to a F atom was established; subsequently, the most stable Na-adsorbed site on this surface was obtained (Fig. 4e). The calculation results show that a Na atom on the most stable site of N-doped Ti_3_C_2_T_*x*_ (− 3.22 eV) has the lower *E*_ads_ than that of pristine Ti_3_C_2_T_*x*_ (− 3.05 eV). This finding easily indicates that the increased N atom reduces the adsorption energy of total system, thereby promoting the Na-ion storage. Importantly, the electrochemical diffusion barriers could intuitively reflect the Na-ion storage kinetics, which is a great point of exploration. Hence, the nudged elastic band (NEB) method was performed to investigate the diffusion barriers of Na ion on the Ti_3_C_2_T_*x*_ surface with or without N-doping. Beforehand, the most stable structures were set as the final sites and the same diffusion paths were controlled (Fig. S13). The obtained result shows that ≈ 0.03 eV energy barrier reduces after introducing a N atom at the expense of a F atom (Fig. 4f). The calculation results prove that the Na-ionic diffusion rate increases after N-doping; subsequently, the N-doped Ti_3_C_2_T_*x*_ surface absorbs Na-ion easier, thereby boosting the additional intercalation of free Na ions from the electrolyte, which is in good agreement with the experimental results.

### Electrochemical Performance Tests in NICs

**Fig. 5:**
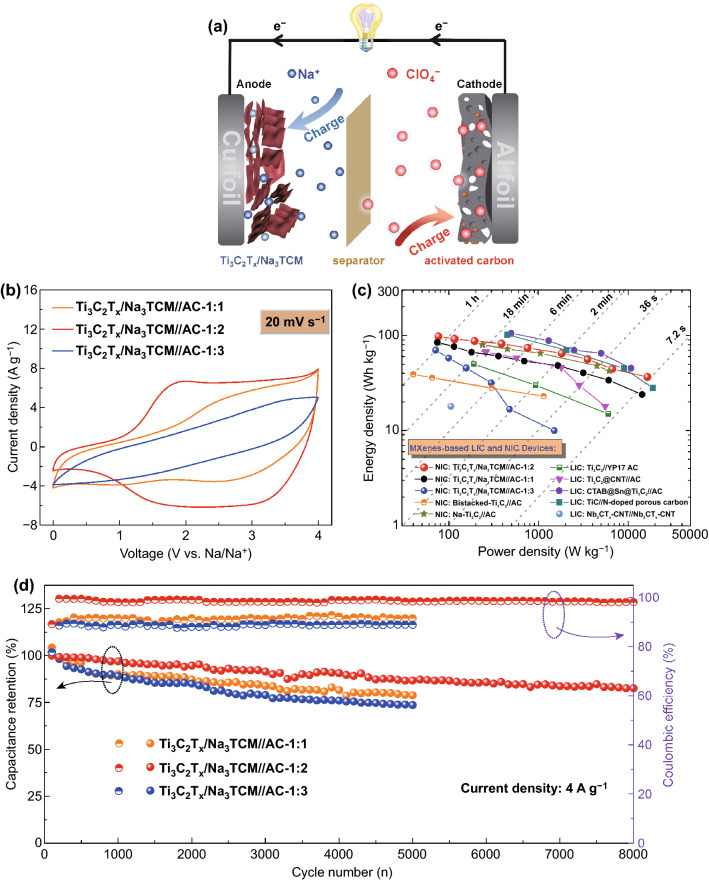
**a** Charge storage mechanism of the Ti_3_C_2_T_*x*_/Na_3_TCM//AC NICs. **b** CV curves of Ti_3_C_2_T_*x*_/Na_3_TCM//AC NICs based on various anode/cathode ratios (1:1, 1:2, 1:3) at a scan rate of 20 mV s^−1^. **c** Ragone plots of the Ti_3_C_2_T_*x*_/Na_3_TCM//AC NICs compared with the advanced MXene-based LICs and NICs. **d** Capacitance retention and coulombic efficiency of the Ti_3_C_2_T_*x*_/Na_3_TCM//AC tested at 4.0 A g^−1^

Basing on the excellent Na-ion storage performance, Ti_3_C_2_T_*x*_/Na_3_TCM was used as anode coupled with the YP80F AC cathode to assemble NICs (Ti_3_C_2_T_*x*_/Na_3_TCM//AC) in the 1 M NaClO_4_ electrolyte. The pore structure characteristics of the AC and the electrochemical performance of AC//Na half-cell within 1.5–4.0 V (vs. Na^+^/Na) are displayed in Figs. S14 and S15, respectively. Figure 5a illustrates the charge storage mechanism of the Ti_3_C_2_T_*x*_/Na_3_TCM//AC NICs. During the charge process, ClO_4_^−^ ions are trapped at the defects and absorbed on the O-contained surface of AC cathode. For the anode, Na^+^ ions are intercalated into the interlayers and absorbed on the N-rich surface of Ti_3_C_2_T_*x*_/Na_3_TCM. Similarly, the discharge process is reversible but reverse. Anode/cathode dynamic matching is the key to optimize performance of hybrid ion capacitors, and the electrode mass ratio is thus optimized to balance the capacities and achieve targeted voltage swings [58]. Basing on the charge balance principle [59], the asymmetric NICs coupling the Ti_3_C_2_T_*x*_/Na_3_TCM anode with AC cathode in the mass ratio of 1:1, 1:2, and 1:3 were constructed. The voltage window of the NICs was set from 0 to 4 V (vs. Na^+^/Na) to maximize the working voltage (Fig. S16). Figure 5b compares the typical CV curves of the above different NIC at a scan rate of 20 mV s^−1^. Apparently, the shapes of these CV curves are diverged from the ideal rectangular shape, indicating the combined contribution of the Faradaic and non-Faradaic reactions. Further, Fig. S17 shows the CV and GC/D curves of the three Ti_3_C_2_T_*x*_/Na_3_TCM//AC NICs at the different rates, which also demonstrate the multiple energy storage mechanisms. It can be easily observed that the Ti_3_C_2_T_*x*_/Na_3_TCM//AC NIC-1:2 has the highest energy storage performance obtained by comparing the integral areas. The energy–power densities of the devices were quantificationally obtained based on the total mass of the active materials of both anode and cathode (Fig. 5c). The Ti_3_C_2_T_*x*_/Na_3_TCM//AC NIC-1:2 shows excellent energy–power densities of 97.6 Wh kg^−1^ at 76 W kg^−1^ and remains 36.6 Wh kg^−1^ even at 16.5 kW kg^−1^, and the maximal power density is higher than the recommended target for commercial electric vehicles (15 kW kg^−1^). It is worth to note that the Ti_3_C_2_T_*x*_/Na_3_TCM//AC NIC-1:2 could finish charge/discharge process within 36 s, and a high energy density of 50 Wh kg^−1^ is still achieved. It is sure that the superior energy–power characteristics of Ti_3_C_2_T_*x*_/Na_3_TCM//AC NIC-1:2 is attributed to the reasonable charge matching, which reduces the ion transport resistance and generates a stable solid–electrolyte interface [58, 59]. In contrast, the energy density of Ti_3_C_2_T_*x*_/Na_3_TCM//AC NIC-1:3 drops sharply as the power density increases, indicating the difference in charge-transfer kinetics at the high-power outputs.

It is instructive to compare the energy–power characteristics of the device to the state-of-the-art reported asymmetric MXene//carbon configurations in the literature. Hence, the Ragone plot of the Ti_3_C_2_T_*x*_/Na_3_TCM//AC NICs was performed in comparison with the advanced MXene-based NICs and LICs, such as Na-Ti_3_C_2_//AC (NIC) [8], bistacked-Ti_3_C_2_//YP17 AC (NIC) [29], Ti_2_C//AC (LIC) [60], Ti_3_C_2_@CNT//AC (LIC) [61], CTAB@Sn@Ti_3_C_2_//AC (LIC) [62], TiC//N-doped porous carbon (LIC) [63], and Nb_2_CT_*x*_-CNT//Nb_2_CT_*x*_-CNT (LIC) [64]. Indeed, the field of asymmetric MXene//carbon capacitors is almost new and contains few examples of complete devices. As could be seen from Fig. 5c, although the Ti_3_C_2_T_*x*_/Na_3_TCM//AC NIC-1:2 not achieves the highest value of energy density compared with the previously reported devices, it is undeniable that this device belongs to one of the best examples with the integrated considerations of energy and power characteristics. Furthermore, the cycling performance of all obtained devices was evaluated at the current density of 4.0 A g^−1^ (Fig. 5d). The Ti_3_C_2_T_*x*_/Na_3_TCM//AC NIC-1:2 exhibits a preeminent capacitance retention of ≈ 90.8% after 4000 cycles and ≈ 82.6% after 8000 cycles. The coulombic efficiency in the range of 97–99% is shown during the steady-state cycling, which is on par with that reported hybrid devices [8, 65]. The low coulombic efficiency shown in the other mass matching may be due to the mismatching of anode and cathode kinetics. In brief, the advanced electrochemical performances of Ti_3_C_2_T_*x*_/Na_3_TCM//AC NIC-1:2 device result from the following noteworthy reasons: (1) The pre-intercalated Na ions pillar the Ti_3_C_2_T_*x*_ interlayer and supply for the irreversible Na ions trapped in the nanovoids; (2) the enlarged surface area and average pore size provide abundant active surface; (3) the in situ formed N atoms significantly increase the pseudocapacitive response; and (4) the reasonable dynamic matching balances the charge of anode and cathode.

## Conclusions

In summary, we have demonstrated a novel and reliable strategy for synthesizing highly N-doped 2D Ti_3_C_2_T_x_ nanosheets and performed their superior Na-ion storage performance. Benefiting from the enlarged interlayer spacing and N-rich surface, the designed Ti_3_C_2_T_x_/Na_3_TCM exhibited fast and high Na-ion storage capability that was verified through joint results of experiments and DFT calculations. With an asymmetric Ti_3_C_2_T_x_ MXene//activated carbon configuration, the 4.0 V Ti_3_C_2_T_*x*_/Na_3_TCM//AC-1:2 NIC device has been successfully assembled, delivering high energy density of 97.6 Wh kg^−1^, high power density of 16.5 kW kg^−1^, and outstanding cycle stability of 82.6% capacitance retention after 8000 cycles. The chemistry of N-doping Ti_3_C_2_T_*x*_ by in situ trimerization strategy employed in this work is available for further studying the competitive polymer molecules/MXenes hybrid electrodes with high electrochemical activity.

## Electronic supplementary material

Below is the link to the electronic supplementary material.

Supplementary file1 (PDF 1544 kb)
