# Supplementary material for: A Novel Strategy of In Situ Trimerization of Cyano Groups Between the Ti3C2Tx (MXene) Interlayers for High-Energy and High-Power Sodium-Ion Capacitors
Source: Nanomicro Lett. 2020 Jun 25;12:135. doi: 10.1007/s40820-020-00473-7 (PMC7770942; doi:10.1007/s40820-020-00473-7)
Supplement: Supplementary file 1 — Supplementary file1 (PDF 1544 kb) [file 40820_2020_473_MOESM1_ESM.pdf]

Supporting Information for

A Novel Strategy of In-Situ Trimerization of Cyano Groups between the $Ti_3C_2T_x$ (MXene) Interlayers for High Energy and Power Sodium-Ion Capacitors

Siyang Liu[1], Fangyuan Hu[1, *], Wenlong Shao[2], Wenshu Zhang[1], Tianpeng Zhang[1], Ce Song[3], Man Yao[1], Hao Huang[1], Xigao Jian[1, 2, 3, *]

[1]School of Materials Science and Engineering, State Key Laboratory of Fine Chemicals, Key Laboratory of Energy Materials and Devices (Liaoning Province), Dalian University of Technology, Dalian 116024, People's Republic of China

[2]State Key Laboratory of Fine Chemicals, Department of polymer Science & Engineering, Dalian University of Technology, Dalian 116024, People's Republic of China

[3]School of Mathematical Sciences, Dalian University of Technology, Dalian 116024, People's Republic of China

*Corresponding authors. E-mail: hufangyuan@dlut.edu.cn (F. Hu), jian4616@dlut.edu.cn (X. Jian)

Supplementary Tables and Figures

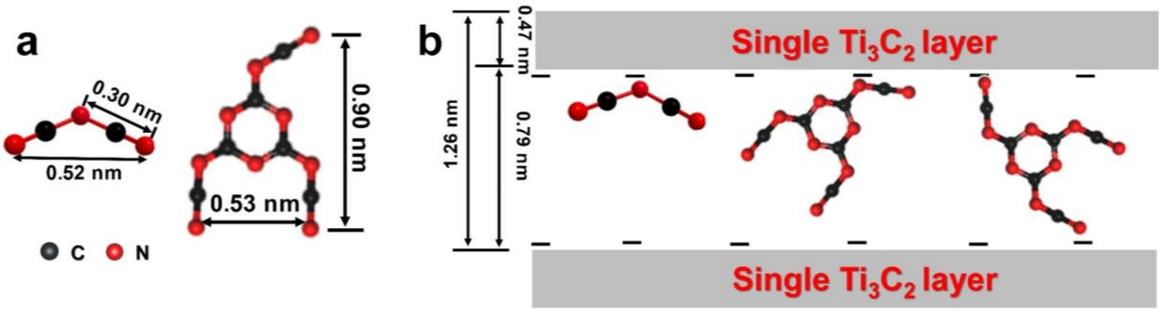

Fig. S1 a Schematic drawing about the structure of dca^- and TCM^{3-} ions. **b** Possible position of dca^- and TCM^{3-} ions in the interlayer of Ti_3C_2 Mxene

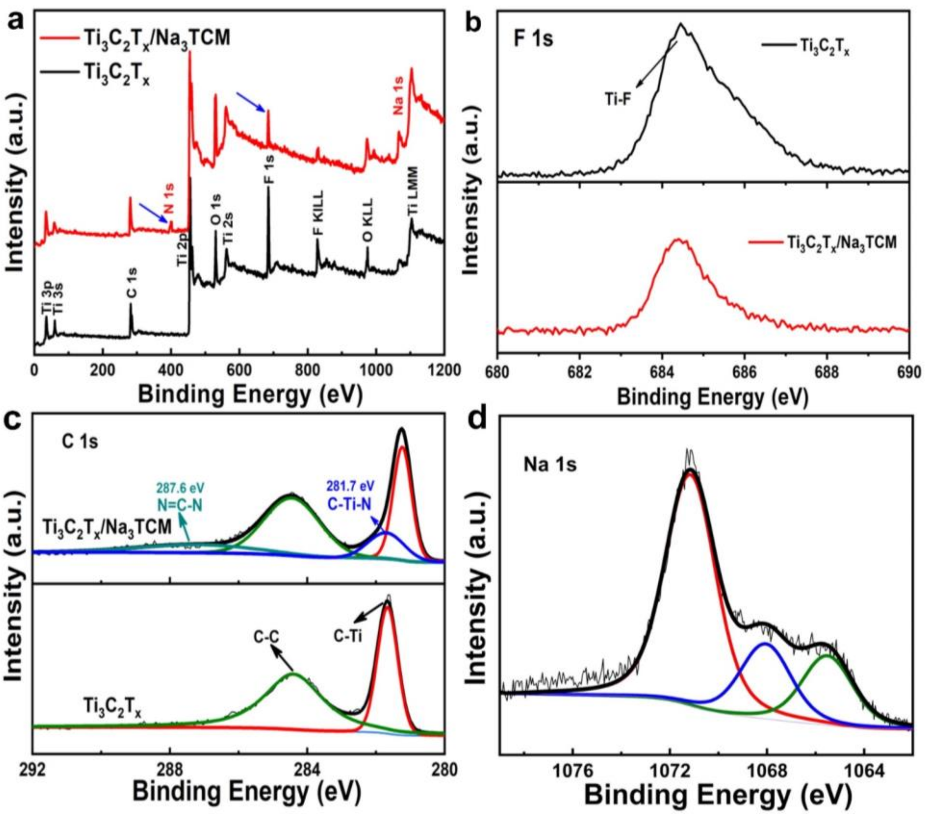

Fig. S2 a XPS survey spectra of $Ti_3C_2T_x/Na_3TCM$ and $Ti_3C_2T_x$. **b** High-resolution F 1s XPS spectra of $Ti_3C_2T_x/Na_3TCM$ (bottom) and $Ti_3C_2T_x$ (top). **c** High-resolution C 1s XPS spectra of $Ti_3C_2T_x/Na_3TCM$ (top) and $Ti_3C_2T_x$ (bottom). **d** High-resolution Na 1s spectrum of $Ti_3C_2T_x/Na_3TCM$

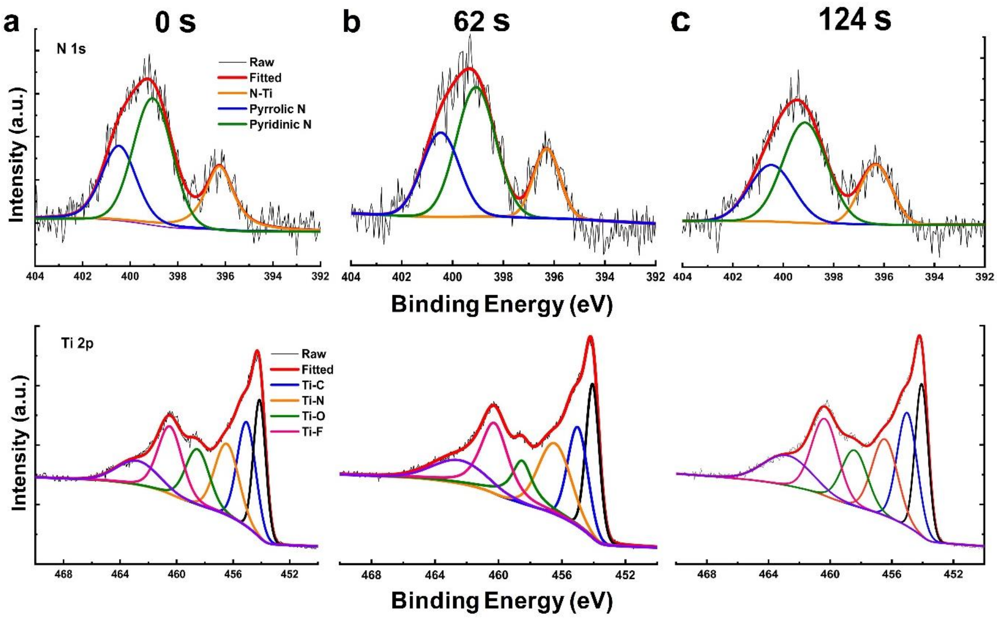

Fig. S3 The depth profiling of XPS characterization for $Ti_3C_2T_x/Na_3TCM$. The high-resolution N 1s and Ti 2p spectra are exhibited in rows and the corresponding results in columns for **a** 0 s, **b** 62 s and **c** 124 s of sputtering

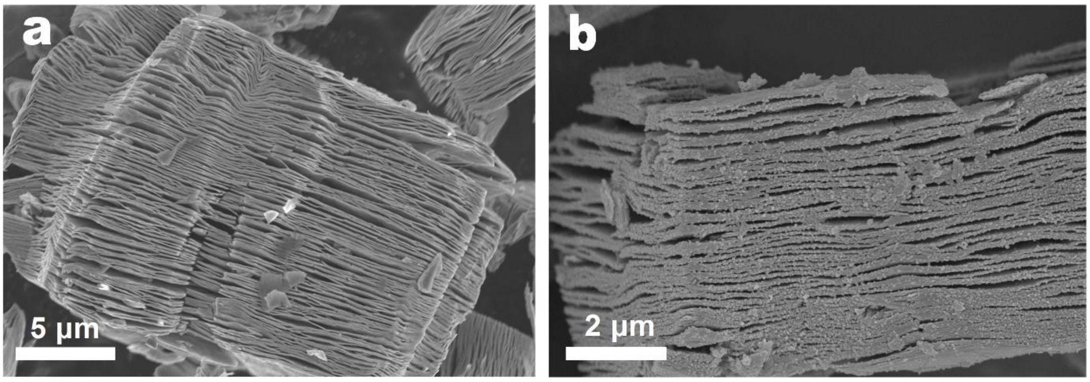

Fig. S4 Cross-sectional SEM images for **a** $Ti_3C_2T_x$ and **b** $Ti_3C_2T_x/Na_3TCM$

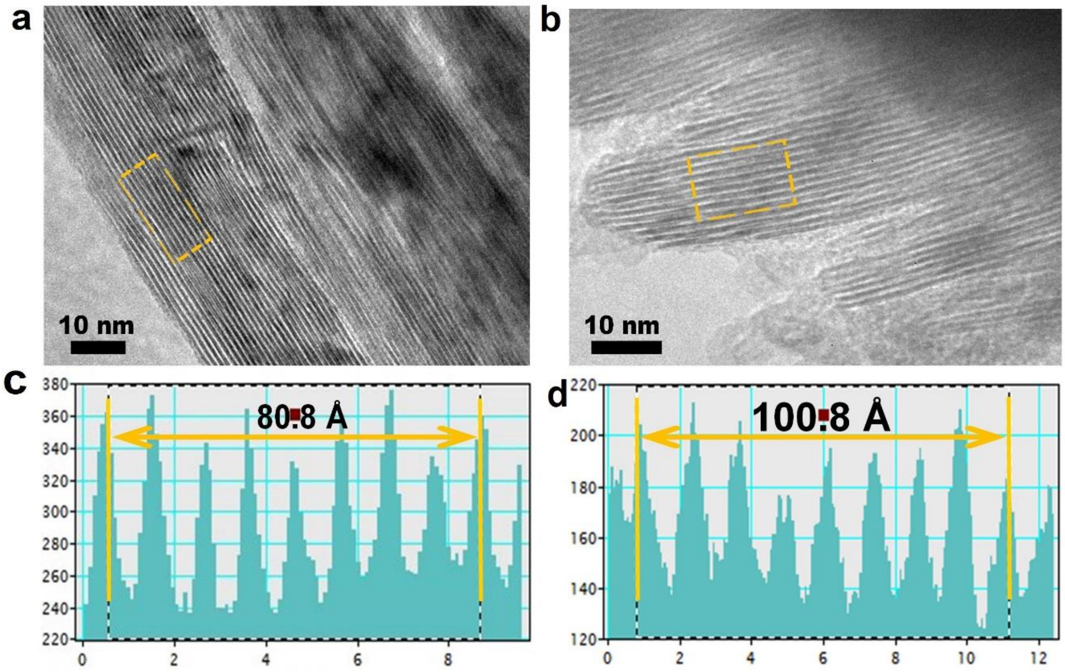

Fig. S5 HR-TEM images and the obtained interlayer spacing patterns of **a, c** $Ti_3C_2T_x$ and **b, d** $Ti_3C_2T_x/Na_3TCM$

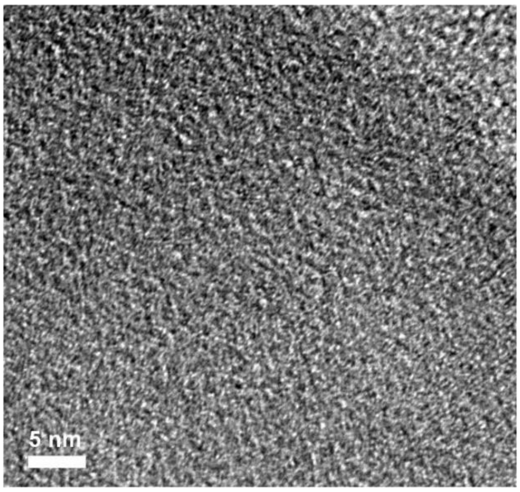

Fig. S6 Aberration-corrected HR-TEM image of the $Ti_3C_2T_x/Na_3TCM$.

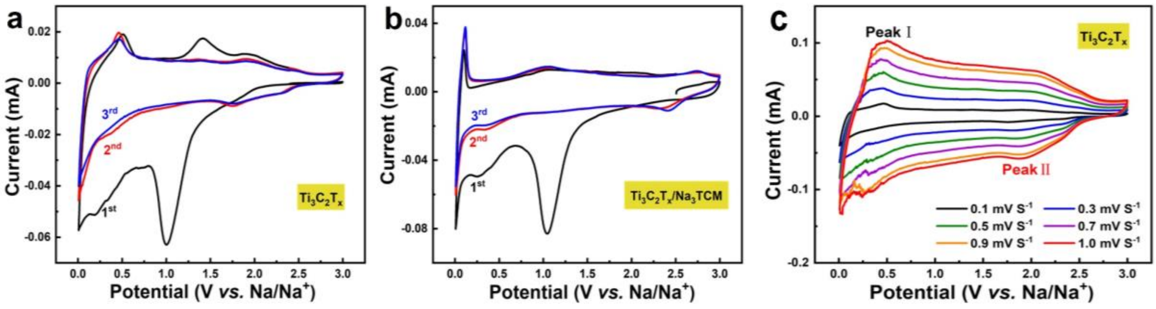

Fig. S7 The current response *vs.* scan rate. **a** The first three CV curves of $Ti_3C_2T_x$ at a scan rate of 0.1 mV s^{-1}. **b** The first three CV curves of $Ti_3C_2T_x/Na_3TCM$ at a scan rate of 0.1 mV s^{-1}. **c** CV curves of $Ti_3C_2T_x$ at the different scan rates

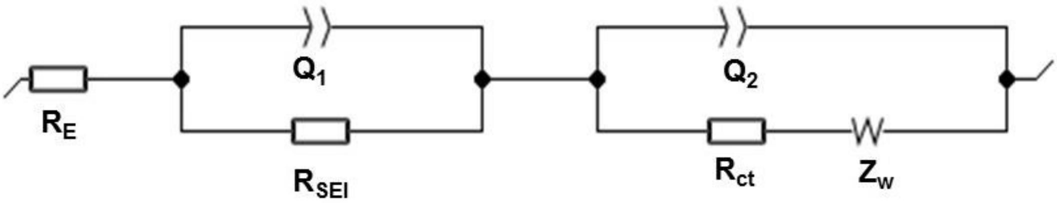

Fig. S8 Equivalent circuit model of Nyquist plots. R_E stands for electrolyte resistance, R_{SEI} stands for SEI layer resistance, R_{ct} stands for changer-transfer resistance, and Z_w is warburg impedance, which accounts for the inclined line

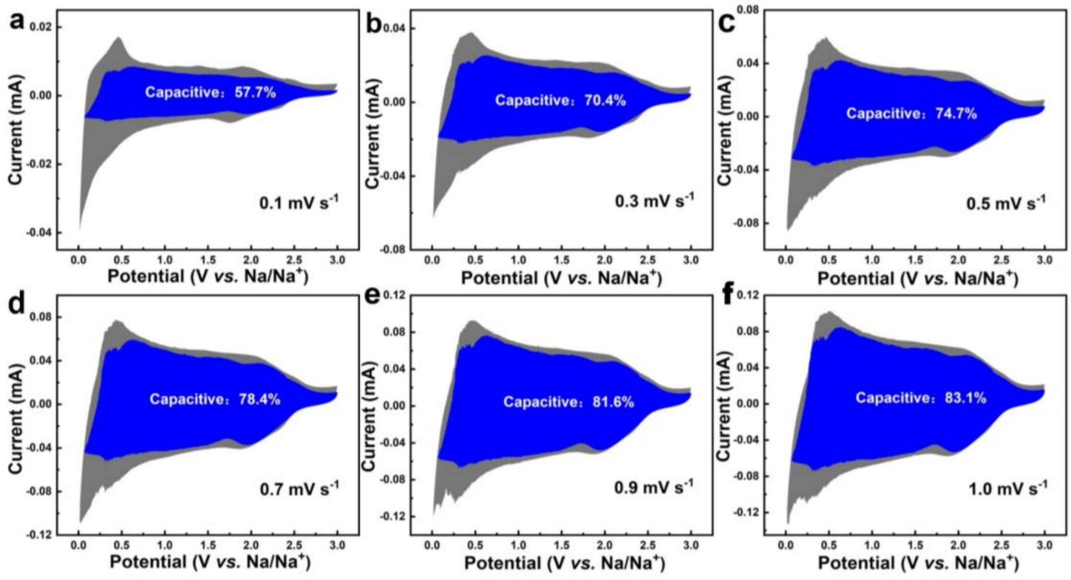

Fig. S9 Kinetics analysis of the Na-ion storage behaviors of $Ti_3C_2T_x$. Separation of the capacitance-controlled (blue region) and diffusion-controlled (gray region) for $Ti_3C_2T_x$. The response of capacitive gradually rises as the scan rate increases of **a** 0.1 mV s^{-1}; **b** 0.3 mV s^{-1}; **c** 0.5 mV s^{-1}; **d** 0.7 mV s^{-1}; **e** 0.9 mV s^{-1}; **f** 1 mV s^{-1}.

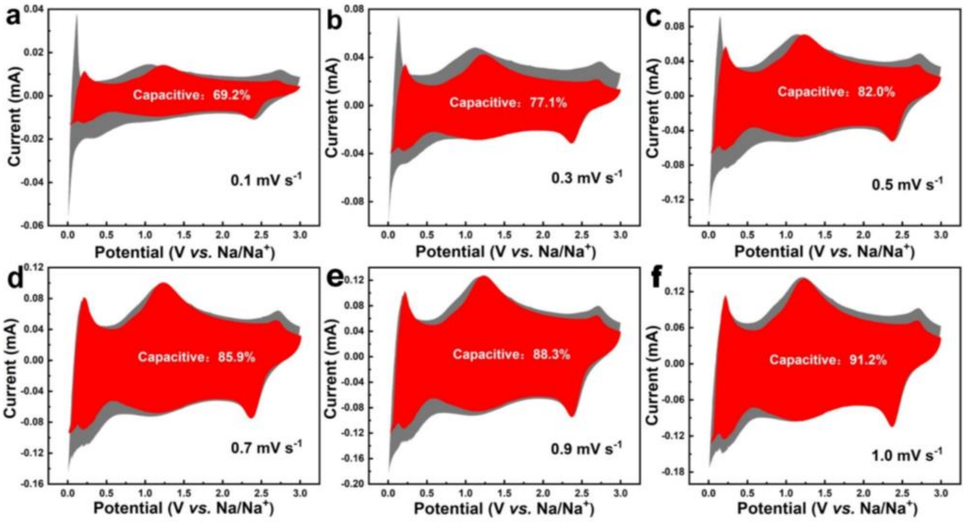

Fig. S10 Kinetics analysis of the Na-ion storage behaviors of $Ti_3C_2T_x/Na_3TCM$. Separation of the capacitance-controlled (red region) and diffusion-controlled (gray region) for $Ti_3C_2T_x$. The response of capacitive gradually rises as the scan rate increases of **a** 0.1 mV s^{-1}; **b** 0.3 mV s^{-1}; **c** 0.5 mV s^{-1}; **d** 0.7 mV s^{-1}; **e** 0.9 mV s^{-1}; **f** 1 mV s^{-1}

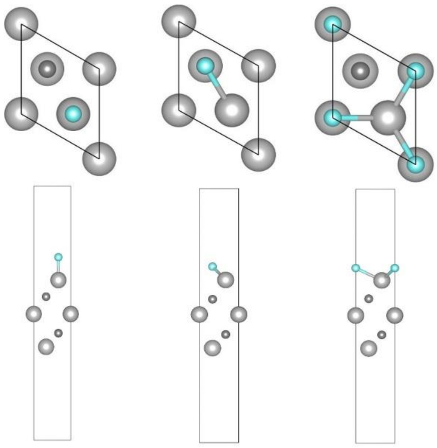

Fig. S11 Top-view and side-view of an O atom adsorbed on 1 × 1 Ti_3C_2 surface at the top-site (left), bcc-site (middle) and fcc-site (right)

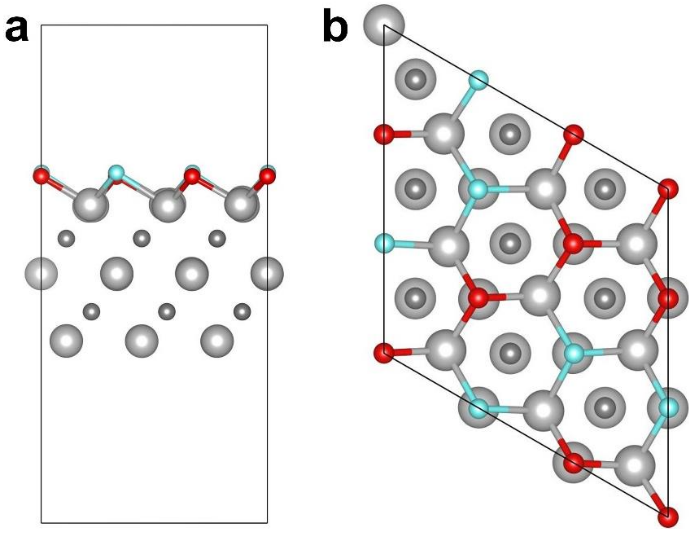

Fig. S12 The Ti_3C_2OF structure model. **a** side-view and **b** top-view of O and F atoms adsorbed on the 3 × 3 Ti_3C_2 surface

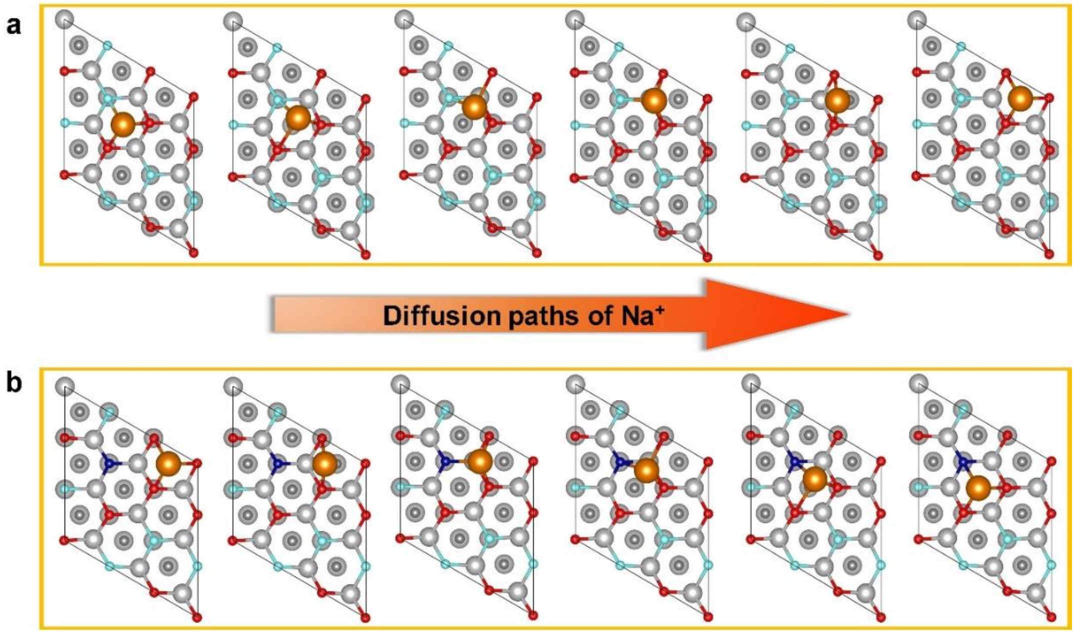

Fig. S13 The diffusion paths of Na-ion on the **a** $Ti_3C_2T_x$ and **b** $Ti_3C_2T_x/Na_3TCM$ surface. As shown by the diffusion path, Na-ion moves to the most stable site, which has the minimum adsorption energy of Na-ions

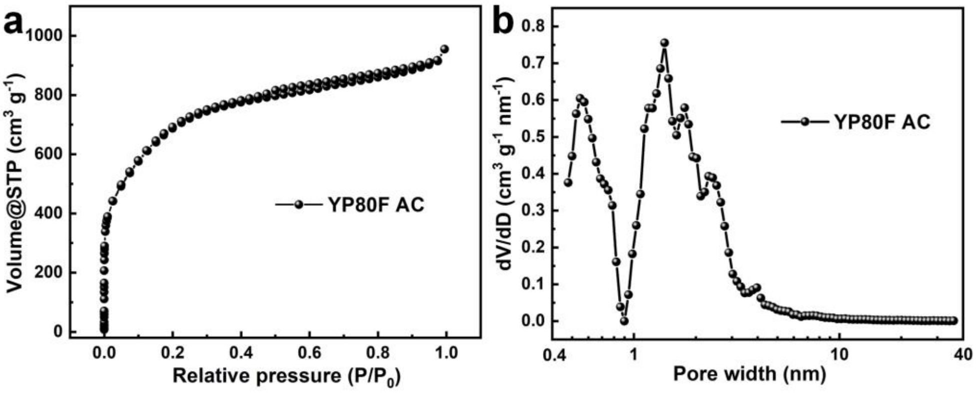

Fig. S14 **a** Nitrogen adsorption-desorption isotherms of the YP80F AC. **b** The pore size distribution of the YP80F AC. The YP80F AC possesses a Brunauer-Emmett-Teller specific surface area of ≈2526 m^2 g^{-1}

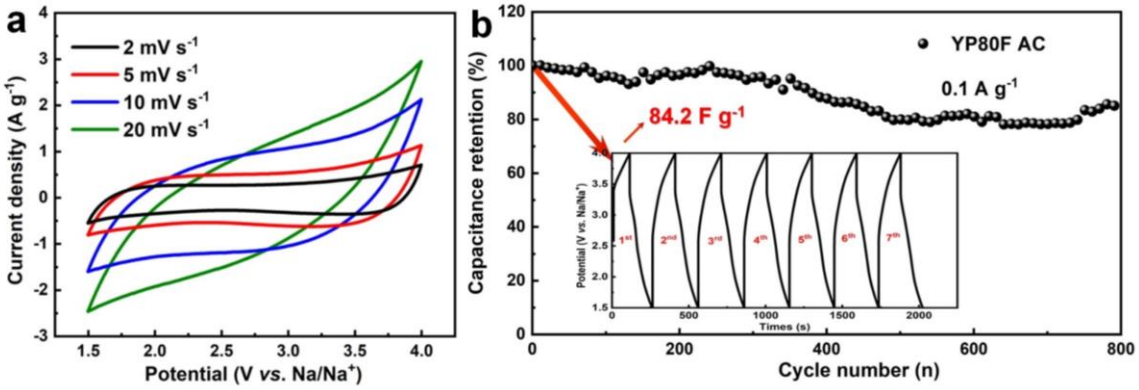

Fig. S15 Half-cell performance of the AC *vs*. Na metal, tested between 1.5−4.0 V. **a** CV curves at different scan rates from 2 mV s^{-1} to 20 mV s^{-1}, **b** Cycling stability at the current density of 0.1 A g^{-1}, insert shows first seven GC/D curves

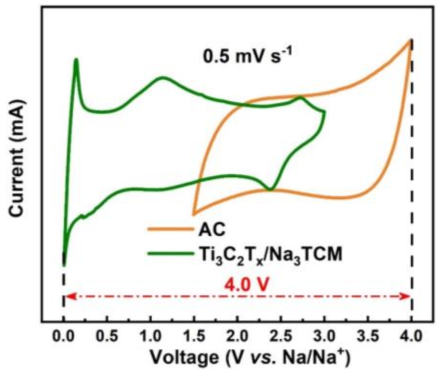

Fig. S16 CV curves of $Ti_3C_2T_x$/Na_3TCM and AC electrodes at the scan rate of 0.5 mV s^{-1}

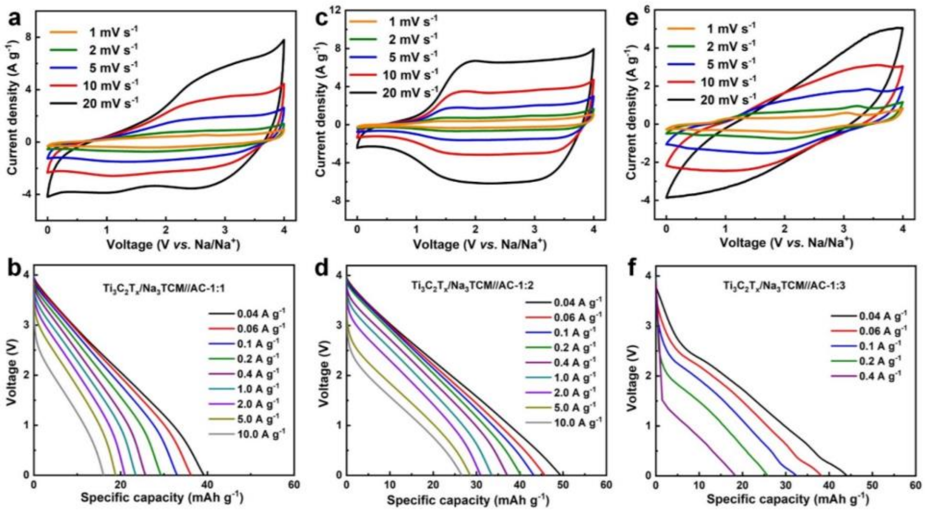

Fig. S17 CV curves of $Ti_3C_2T_x$/Na_3TCM//AC NICs in different anode/cathode mass ratios: **a** 1:1; **c** 1:2; **e** 1:3. GC/D profiles of $Ti_3C_2T_x$/Na_3TCM//AC NICs in different anode/cathode mass ratios: **b** 1:1; **d** 1:2; **e** 1:3

Table S1 The XPS elements measurement of $Ti_3C_2T_x$ and $Ti_3C_2T_x$/Na_3TCM

Samples	C (at%)	Ti (at%)	O (at%)	F (at%)	N (at%)	Na (at%)
$Ti_3C_2T_x$	35.3	29.4	19.8	15.5	0.0	0.0
$Ti_3C_2T_x$/Na_3TCM	40.5	24.2	21.1	5.2	5.6	3.4

Table S2 Impedance parameters of $Ti_3C_2T_x$−1, $Ti_3C_2T_x$−50, $Ti_3C_2T_x$−200 and $Ti_3C_2T_x$/Na_3TCM−1, $Ti_3C_2T_x$/Na_3TCM−50, $Ti_3C_2T_x$/Na_3TCM−200

Samples	R_E (ohm)	R_{SEI} (ohm)	R_{ct} (ohm)
$Ti_3C_2T_x$−1	4.35	65.69	170.4
$Ti_3C_2T_x$−50	4.92	74.42	223.5
$Ti_3C_2T_x$−200	5.23	336.10	365.9
$Ti_3C_2T_x$/Na_3TCM−1	4.63	68.55	6.25
$Ti_3C_2T_x$/Na_3TCM−50	3.90	68.90	21.81
$Ti_3C_2T_x$/Na_3TCM−200	4.30	93.00	172.10

Table S3 The adsorption energy (E_{ads}) of O and F atom adsorbed on 1 × 1 Ti_3C_2 surface at the top-site, bcc-site and fcc-site

Adsorption sites	top-site	bcc-site	fcc-site
ΔE_{ads} (Ti_3C_2+O)/eV	-7.36	-8.98	-9.74
ΔE_{ads} (Ti_3C_2+F)/eV	-6.42	-6.80	-7.20

Table S4 Summary of electrochemical performance for the pseudocapacitive oxides and MXenes-based LIC and NIC devices

device configuration (anode//cathode)	type	Voltage window	max energy density (Wh/kg)/max power density (W/kg)	capacity retention
Nb_2CT_x-CNT//$LiFePO_4$ [S1]	LIC	0−3 V	–	69.5% over 500 cycles
T-Nb_2O_5@C//MSP-20 AC [S2]	LIC	1−3.5 V	63/6500	75% over 1K cycles
Ti_2C-MXene //YP17 AC [S3]	LIC	1−3.5 V	50/600	85% over 1K cycles
CTAB-Sn@Ti_3C_2//AC [S4]	LIC	1−4 V	105.6/10800	70% over 4K cycles
TiC-MXene//N-doped porous carbon [S5]	LIC	0−4.5 V	101.5/67500	82% over 5K cycles
Nb_2O_5 nanosheets//AC [S6]	NIC	1−3 V	43.2/5760	80% over 3K cycles
V_2O_5@CNT//AC [S7]	NIC	0−2.8 V	38/5000	80% over 0.9K cycles
Na-Ti_3C_2//AC [S8]	NIC	1−3.75V	80/6172	78.4% over 15K cycles
Ti_3C_2 MXene-CNT//$Na_{0.44}MnO_2$ [S9]	NIC	0−4 V	–	90% over 60 cycles
Bistacked-Ti_3C_2//AC [S10]	NIC	0.6-4 V	39/1140	84% over 4K cycles
$Ti_3C_2T_x$/Na_3TCM//AC-1:1 (this work)	NIC	0−4 V	84.3/14382	78.8% over 5K cycles
$Ti_3C_2T_x$/Na_3TCM//AC-1:3 (this work)	NIC	0−4 V	70.1/1500	73.6% over 5K cycles
**$Ti_3C_2T_x$/Na_3TCM//AC-1:2 (this work)**	**NIC**	**0−4 V**	**97.6/16481**	**82.6% over 8K cycles**

Supplementary References

[S1] A. Byeon, A.M. Glushenkov, B. Anasori, P. Urbankowski, J. Li et al., Lithium-ion capacitors with 2D Nb_2CT_x (MXene)-carbon nanotube electrodes. J. Power Sources **326**, 686−694 (2016). https://doi.org/10.1016/j.jpowsour.2016.03.066

[S2] E. Lim, C. Jo, H. Kim, M.-H. Kim, Y. Mun et al., Facile synthesis of Nb_2O_5@carbon core-shell nanocrystals with controlled crystalline structure for high-power anodes in hybrid supercapacitors. ACS Nano **9**, 7497−7505 (2015). https://doi.org/10.1021/acsnano.5b02601

[S3] J. Come, M. Naguib, P. Rozier, M.W. Barsoum, Y. Gogotsi et al., A non-aqueous asymmetric cell with a Ti_2C-based two-dimensional negative electrode. J. Electrochem. Soc. **159**, A1368−A1373 (2012). https://doi.org/10.1149/2.003208jes

[S4] J. Luo, W. Zhang, H. Yuan, C. Jin, L. Zhang et al., Pillared structure design of MXene

with ultralarge interlayer spacing for high-performance lithium-ion capacitors. ACS Nano **11**, 2459−2469 (2017). https://doi.org/10.1021/acsnano.6b07668

[S5] H. Wang, Y. Zhang, H. Ang, Y. Zhang, H.T. Tan et al., A high-energy lithium-ion capacitor by integration of a 3D interconnected titanium carbide nanoparticle chain anode with a pyridine-derived porous nitrogen-doped carbon cathode. Adv. Funct. Mater. **26**, 3082−3093 (2016). https://doi.org/10.1002/adfm.201505240

[S6] H. Li, Y. Zhu, S. Dong, L. Shen, Z. Chen et al., Self-assembled Nb_2O_5 nanosheets for high energy-high power sodium ion capacitors. Chem. Mater. **28**, 5753−5760 (2016). https://doi.org/10.1021/acs.chemmater.6b01988

[S7] Z. Chen, V. Augustyn, X. Jia, Q. Xiao, B. Dunn, Y. Lu, High-performance sodium-ion pseudocapacitors based on hierarchically porous nanowire composites. ACS Nano **6**, 4319−4327 (2012). https://doi.org/10.1021/nn300920e

[S8] J. Luo, C. Fang, C. Jin, H. Yuan, O. Sheng et al., Tunable pseudocapacitance storage of MXene by cation pillaring for high performance sodium ion capacitors. J. Mater. Chem. A **6**, 7794−7806 (2018). https://doi.org/10.1039/c8ta02068j

[S9] X. Xie, M.-Q. Zhao, B. Anasori, K. Maleski, C.E. Ren et al., Porous heterostructured MXene/carbon nanotube composite paper with high volumetric capacity for sodium-based energy storage devices. Nano Energy **26**, 513−523 (2016). https://doi.org/10.1016/j.nanoen.2016.06.005

[S10] N. Kurra, M. Alhabeb, K. Maleski, C.-H. Wang, H.N. Alshareef, Y. Gogotsi, Bistacked titanium carbide (MXene) anodes for hybrid sodium ion capacitors. ACS Energy Lett. **3**, 2094−2100 (2018). https://doi.org/10.1021/acsenergylett.8b01062
